# Osteoporosis-related fracture case definitions for population-based administrative data

**DOI:** 10.1186/1471-2458-12-301

**Published:** 2012-05-18

**Authors:** Lisa M Lix, Mahmoud Azimaee, Beliz Acan Osman, Patricia Caetano, Suzanne Morin, Colleen Metge, David Goltzman, Nancy Kreiger, Jerilynn Prior, William D Leslie

**Affiliations:** 1 School of Public Health, University of Saskatchewan, Saskatoon, Canada; 2 University of Manitoba, Winnipeg, Canada; 3 Institute for Clinical Evaluative Sciences, Toronto, Canada; 4 Health Quality Council, Saskatoon, Canada; 5 McGill University, Montreal, Canada; 6 University of Toronto, Toronto, Canada; 7 University of British Columbia, Vancouver, Canada

## Abstract

**Background:**

Population-based administrative data have been used to study osteoporosis-related fracture risk factors and outcomes, but there has been limited research about the validity of these data for ascertaining fracture cases. The objectives of this study were to: (a) compare fracture incidence estimates from administrative data with estimates from population-based clinically-validated data, and (b) test for differences in incidence estimates from multiple administrative data case definitions.

**Methods:**

Thirty-five case definitions for incident fractures of the hip, wrist, humerus, and clinical vertebrae were constructed using diagnosis codes in hospital data and diagnosis and service codes in physician billing data from Manitoba, Canada. Clinically-validated fractures were identified from the Canadian Multicentre Osteoporosis Study (CaMos). Generalized linear models were used to test for differences in incidence estimates.

**Results:**

For hip fracture, sex-specific differences were observed in the magnitude of under- and over-ascertainment of administrative data case definitions when compared with CaMos data. The length of the fracture-free period to ascertain incident cases had a variable effect on over-ascertainment across fracture sites, as did the use of imaging, fixation, or repair service codes. Case definitions based on hospital data resulted in under-ascertainment of incident clinical vertebral fractures. There were no significant differences in trend estimates for wrist, humerus, and clinical vertebral case definitions.

**Conclusions:**

The validity of administrative data for estimating fracture incidence depends on the site and features of the case definition.

## Background

Osteoporosis is a common condition amongst older adults. It is estimated to affect up to 20% of post-menopausal women [1]. Monitoring the incidence of osteoporosis-related fractures is therefore an important component of a comprehensive population health surveillance system [2-4]. Administrative data, routinely collected records used for health system management and physician remuneration, are a common tool for population-based studies about infectious and chronic conditions because these data contain diagnosis codes for ascertaining disease cases. Administrative data also contain diagnosis codes to ascertain fracture cases and have been used in many countries to study trends in osteoporosis-related fracture rates [5,6] and risk factors [7,8], and to conduct pharmaco-epidemiologic investigations about fracture outcomes [9-11].

The validation studies conducted to date suggest that administrative data are generally sensitive and specific for ascertaining cases of hip fracture [12], although this will vary with the type of hip fracture [13]. Administrative data may lack sensitivity for ascertaining cases of incident clinical vertebral fracture [14,15]. There has been limited research about the validity of administrative data for ascertaining cases of other osteoporosis-related fractures, including fractures of the wrist and humerus [15,16] . As well, characteristics of the fracture case definition that may affect the validity of administrative data for case ascertainment warrant further investigation. Curtis et al. [14] reported that an incident clinical vertebral fracture case definition based on hospital diagnoses had a positive predictive value (PPV) of 91%. A case definition based on diagnoses in a broad set of administrative databases, including hospital, physician billing, and emergency room data, had a PPV of only 46%. Their recommended case definition, which was based on a diagnosis in physician data in combination with a spinal imaging test or a diagnosis in hospital data, had a PPV of 61%. Case definition characteristics that have varied across studies include the administrative data source, diagnosis codes, use of codes for fracture-related services or procedures, and the length of the fracture-free period to ascertain incident cases [17-19].

The purpose of this study was to investigate administrative data case definitions for osteoporosis-related fractures. The specific objectives were to: (a) compare the incidence estimates obtained from administrative data with estimates obtained from population-based clinically-validated data, and (b) test for differences in the incidence estimates from multiple administrative data case definitions.

## Methods

### Data sources

Administrative data were from the central Canadian province of Manitoba, which has a population of approximately 1.2 million. Like other Canadian provinces, Manitoba has a system of universal healthcare. The Manitoba Centre for Health Policy Research Data Repository houses computerized databases that contain records of virtually all contacts with the health care system. The databases can be linked via an anonymized personal identification number. Ethics approval for this research was received from the Manitoba Health Research Ethics Board and permission to access the study data was provided by the Manitoba Health Information Privacy Committee.

Hospital and physician billing claims data were used to construct the fracture case definitions. Diagnoses in hospital data are recorded using the International Classification of Diseases, 9^th^ revision, Clinical Modification (i.e., ICD-9-CM), up to and including the 2003/04 fiscal year (a fiscal year runs from April 1 to March 31) and the International Classification of Diseases, 10^th^ revision, Canadian version (i.e., ICD-10-CA), for subsequent years. A maximum of 16 ICD-9-CM codes and 25 ICD-10-CA codes are recorded on each record. Physicians who are paid on a fee-for-service basis submit billing claims to the provincial health ministry; these claims capture almost all outpatient services, including those provided in hospital emergency and outpatient departments. While a small percentage of physicians are salaried (i.e., about 7% of family physicians [20], approximately 90% submit parallel billing claims for administrative purposes. Physician billing claims contain a single ICD-9-CM code as well as service codes, which are defined in a fee schedule (manitoba.ca/health/manual). Diagnosis codes in hospital and physician data have been validated and used extensively in Manitoba [21-23] and in other Canadian jurisdictions [24] for research about chronic conditions, and they have been used in multiple studies about osteoporosis-related fractures [9,11,25].

Validation data were from the Canadian Multicentre Osteoporosis Study (CaMos) [26]. CaMos is an ongoing, population-based cohort study designed to provide national estimates of the prevalence and incidence of osteoporosis and osteoporosis-related fractures. The study population is composed of non-institutionalized individuals who reside within a 50-km radius of one of nine study centres located across Canada. These geographic areas encompass approximately 40% of the Canadian population and include both rural and urban residents. Households in each area were selected by random draws of listed telephone numbers; one randomly selected household member greater than 25years was asked to participate. A total of 9423 participants entered the study (2884 males and 6539 females) during the 18-month recruitment period. The characteristics of study participants have been described previously [27,28]. A refusal questionnaire was used to assess selection bias. For osteoporosis prevalence, the primary study outcome, selection bias was not observed in any of the study age groups, with the exception of a small amount of bias in the 80+ age group [29]. Informed consent was obtained from participants and the study received approval from the institutional review board at each participating centre. Data on incident fractures was compiled from CaMos participants for the period from 1996 to 2006. These data were collected using annual postal questionnaires and/or in-person interviews [27]. A detailed interviewer-administered questionnaire was used at years 3, 5, and 10. At baseline, year 5, and year 10, lateral lumbar and thoracic spine X-rays and bone mineral density tests were performed. At years 1, 2, 4, and 6 through 9, a detailed two-page questionnaire was mailed to participants asking about hospitalizations and fractures within the past year. Participants who reported having a fracture were asked to provide consent for study staff to collect additional data about the fracture diagnosis from the treating physician and/or hospital record (i.e., verification by radiology report). We limited the study to fractures occurring in individuals 50years of age and older, with age defined at the fracture index date.

### Fracture case definitions

Table 1 lists the 35 administrative data case definitions that were selected for investigation. These were selected based on a review of published studies [3,12,30], recommendations from clinical co-investigators with expertise in fracture ascertainment in administrative databases (WDL, SM), and the authors’ previous experience ascertaining other chronic diseases, include osteoporosis, in administrative data [31-33]. The case definitions were differentiated by: (a) source of data, (b) number of records with the relevant diagnosis code(s), (c) type of diagnosis in hospital data, (d) presence of service codes in physician billing claims, and (e) duration of the fracture-free period. With the exception of one hip fracture case definition, all site-specific definitions used the same ICD-9-CM and ICD-10-CA diagnosis code(s). For hip fracture, we considered ICD-9-CM 820 (fracture of neck of the femur) and 821 (fracture and other unspecified parts of the femur) because some hip fractures may be assigned a less precise diagnosis code [34]. Case definitions were based on hospital data only (hip) or hospital and physician claims data, in keeping with previous research [3,15]. For the latter, case definitions requiring one or at least two records with the specified diagnosis code(s) were considered. Service codes capture radiologic and magnetic resonance imaging services for incident clinical vertebral fracture, immobilization or fixation services for wrist fracture, and surgical repair and fixation procedures for hip fracture. Service codes have also been used in previous studies to improve fracture ascertainment [35]. Fracture-free periods of zero, six or twelve months were considered, using the site-specific fracture index date to establish the end-point of the fracture-free period.

**Table 1:** Osteoporosis-related fracture case definitions

Case def	# of DX & Data source	DX codes	MR versus any DX	Service codes	Fracture-free period (months)
Hip					
H1	1H	ICD-9: 820‒821; ICD-10: S72.0-S72.2	MR	No	0
H2	1H	ICD-9: 820; ICD-10: S72.0-S72.2	MR	No	0
H3	1H	same as H2	MR	No	6
H4	1H	same as H2	MR	No	12
H5	1H	same as H2	Any	No	0
H6	1H	same as H2	Any	No	6
H7	1H	same as H2	Any	No	12
H8	1H	same as H2	MR	Yes	0
H9	1H	same as H2	MR	Yes	6
H10	1H	same as H2	MR	Yes	12
H11	1H	same as H2	Any	Yes	0
H12	1H	same as H2	Any	Yes	6
H13	1H	same as H2	Any	Yes	12
Wrist					
W1	1H or 1 P	ICD-9: 813; ICD-10:S52	Any	Yes	0
W2	1H or 1 P	same as W1	Any	Yes	6
W3	1H or 1 P	same as W1	Any	No	6
W4	1H or (2+ P in 90days)	same as W1	Any	Yes	6
W5	1H or (2+ P in 90days)	same as W1	Any	No	6
W6	1H or (2+ P in 90days)	same as W1	MR	Yes	6
W7	1H or (2+ P in 90days)	same as W1	MR	No	6
Humerus					
U1	1H or 1 P	ICD-9: 812; ICD-10:S42.2	MR	No	0
U2	1H or 1 P	same as U1	MR	No	6
U3	1H or 1 P	same as U1	Any	No	0
U4	1H or 1 P	same as U1	Any	No	6
U5	1H or (2+ P in 90days)	same as U1	MR	No	6
U6	1H or (2+ P in 90days)	same as U1	Any	No	6
U7	1H or (2+ P in 90days)	same as U1	Any	No	12
Clinical vertebral					
V1	1H	ICD-9: 805; ICD-10: S22.0, S22.1, S32.0	MR	No	0
V2	1H	same as V1	Any	No	0
V3	1H or 1 P	same as V1	MR	Yes	0
V4	1H or 1 P	same as V1	Any	No	0
V5	1H or 1 P	same as V1	Any	No	12
V6	1H or 1 P	same as V1	MR	No	12
V7	1H or (2+ P in 90days)	same as V1	MR	No	12
V8	1H or (2+ P in 90days)	same as V1	MR	No	12

Note: DX=diagnosis; H=hospital record; P=physician billing claim; MR=most responsible or primary diagnosis in hospital records; A listing of the service codes used for incident fracture ascertainment is provided in the Appendix.

To illustrate the interpretation of the case definitions, H1 identifies hip fractures using hospital records with ICD-9-CM 820 or 821 (ICD-10-CA S72.0, S72.1, or S72.2) as the most responsible (i.e., primary) diagnosis; it does not use physician service codes nor does it require a fracture-free period. In contrast, case definition H13 identifies hip fractures from hospital records with ICD-9-CM 820 (ICD-10-CA S72.0, S72.1, or S72.2) in any diagnosis field. A physician service code was present within the hospitalization period and a 12-month fracture-free period was adopted. For wrist fracture, case definition W1 identifies fractures using hospital or physician billing records with ICD-9-CM 813 (ICD-10-CA S52) in any diagnosis field. This case definition requires a physician service code to accompany the diagnosis code and does not adopt a fracture-free period.

The fracture index date was the date of the first diagnosis or service code for a fracture event. Pathologic fractures were included because they represent a small proportion of all fractures and their exclusion can lead to underestimation of the fracture burden due to osteoporosis [36]. For each case definition, the number of incident fractures was generated for the Manitoba population 50years of age and older for fiscal years 1997/98 to 2006/07. Age, which was defined using the fracture index date, was obtained from health insurance registration files. For hip fracture, counts of incident fractures were generated both including and excluding residents of long-term care (i.e., nursing home) facilities [37]; the CaMos data excludes residents of these facilities and this may affect comparability of estimates. Residence in a facility was determined from nursing home files containing admission and separation dates.

### Statistical analyses

Crude and sex- and age-specific fracture rates were calculated for administrative case definitions and for the CaMos data. Generalized linear models (GLMs) were used to test for differences in the estimates from administrative and CaMos data [38]. For each case definition, sex-specific models that contained the main effects of age (five-year groupings from 50‒54years to 80+ years) and source (i.e., administrative; reference: CaMos) were fit to the data, along with a model that combined the data for males and females; this latter model contained the main effects of age, source, and sex. A negative binomial distribution was adopted to model the data due to the presence of extra-Poisson variation in the case counts. Fit was evaluated using the ratio of model deviance to degrees of freedom, which should be close to one for a well-fitted model. A Wald ^2^ statistic was used to test the difference in the estimates from administrative and CaMos data. Each test was conducted at the =.01 significance level in order to control the familywise error rate, the probability of committing at least one Type I error. The relative rate (RR) of incident fractures for administrative data case definitions was obtained by exponentiation of the regression coefficient for the source variable.

GLMs with generalized estimating equations were used to test for differences among the case definitions [39]. Annual fracture counts (i.e., for 1997/98 to 2006/07) for population strata defined by age (five-year groupings from 50‒54years to 85+ years) and sex were modeled assuming a negative binomial distribution. An exchangeable structure, which assumes constant correlation in successive years, accounted for dependence among the incident fracture counts. Models containing the main effects of age group, case definition, sex, year, and the year x case definition interaction were fit to the data for each fracture site. The interaction was used to test for differences in the linear trend across case definitions. If the interaction was statistically significant, linear contrasts were used to test for differences amongst selected pairs of case definitions. A significance level of =.05 was adopted for the omnibus test and each of the contrast tests was conducted at =.01. If the interaction was not significant, the main effects of year and case definition were investigated.

## Results

Age-specific and overall fracture incidence estimates from the CaMos data are reported for males and females in Table 2. There were 65905 person years of observation and 875 clinically-recognized incident osteoporosis-related fractures verified in the study period. Of this number, 22.6% were hip, 41.3% were wrist, 13.6% were humerus, and 22.5% were clinical vertebral fractures.

**Table 2:** CaMos Osteoporosis-related incident fracture rates per 100,000 person years (95% confidence intervals)

Age group	Males	Females
			Hip
50-59	33.0 (0.0, 97.6)	29.4 (0.0, 79.1)	
60-69	132.4 (31.5, 233.3)	63.4 (0.0, 159.7)	
70-79	239.1 (116.0, 362.3)	309.6 (230.4, 388.9)	
80+	950.2 (717.2, 1183.2)	1079.1 (928.1, 1230.1)	
All Ages	272.6 (143.5, 401.6)	310.9 (54.0, 567.8)	
			Wrist
50-59	197.9 (39.7, 356.1)	308.6 (147.7, 469.5)	
60-69	115.9 (21.5, 210.3)	615.4 (316.6, 914.3)	
70-79	223.2 (104.2, 342.2)	855.7 (724.3, 914.3)	
80+	228.1 (113.5, 342.6)	763.3 (636.0, 890.5)	
All Ages	183.6 (77.6, 289.5)	684.4 (303.9, 1064.8)	
			Humerus
50-59	33.0 (0.0, 97.6)	29.4 (0.0, 79.1)	
60-69	82.8 (3.0, 162.6)	184.0 (20.2, 347.8)	
70-79	95.6 (17.7, 173.6)	242.1 (172.0, 312.2)	
80+	228.1 (113.5, 342.6)	644.8 (527.8, 761.8)	
All Ages	100.1 (21.9, 178.4)	256.6 (23.2, 490.1)	
			Clinical vertebral
50-59	164.9 (20.5, 309.3)	176.3 (54.7, 298.0)	
60-69	115.9 (21.5, 210.3)	152.3 (3.3, 301.3)	
70-79	207.2 (92.6, 321.9)	394.1 (304.7, 483.4)	
80+	304.1 (171.8, 436.3)	763.3 (636.0, 890.5)	
All Ages	183.6 (77.6, 289.5)	342.2 (72.7, 611.7)	

In the Manitoba population in the 10-year period, there were more than three million person years of observation. RR estimates and tests of differences in adjusted incidence estimates for the CaMos and administrative data are reported in Table 3. For hip fractures, when the data for both sexes were combined and long-term care residents were included, case definitions H5, H6, and H7 resulted in RR estimates that were significantly greater than the CaMos estimates (i.e., over-ascertainment), while the remaining case definitions did not result in significantly different estimates. For females, all case definitions except for H8, H9, and H10 resulted in over-ascertainment of hip fracture rates. For males, these three case definitions resulted in RR estimates that were significantly lower than the CaMos estimates (i.e., under-ascertainment).

**Table 3:** Relative Rates (RRs) of osteoporosis-related incident fractures in administrative data using CaMos as the reference

Case def			Both sexes			Females			Males
	RR	*p*-value	RR	*p*-value	RR	*p*-value			
							Hip
H1	1.21	.0318	**1.40**	<.0001	0.89	.4295	
H2	1.14	.1408	**1.31**	.0008	0.85	.2542	
H3	1.11	.2567	**1.27**	.0028	0.83	.1931	
H4	1.10	.2963	**1.26**	.0045	0.82	.1813	
H5	**1.50**	<.0001	**1.73**	<.0001	1.12	.4481	
H6	**1.45**	<0001	**1.67**	<.0001	1.08	.5837	
H7	**1.43**	<0001	**1.64**	<.0001	1.07	.5235	
H8	0.94	.4874	1.08	.3160	**0.68**	.0083	
H9	0.93	.4322	1.07	.3837	**0.68**	.0074	
H10	0.93	.3863	1.06	.4626	**0.68**	.0069	
H11	1.23	.0167	**1.42**	<.0001	0.89	.4112	
H12	1.22	.0205	**1.41**	<.0001	0.88	.3887	
H13	1.21	.0238	**1.39**	<.0001	0.88	.3695	
							Wrist
W1	1.15	.0731	1.17	.0055	1.06	.7176	
W2	**0.69**	<.0001	**0.71**	<.0001	0.66	.0162	
W3	1.02	.7420	1.03	.6599	1.06	.7502	
W4	**0.62**	<.0001	**0.63**	<.0001	**0.58**	.0015	
W5	**0.77**	.0005	**0.78**	<.0001	0.73	.0671	
W6	**0.56**	<.0001	**0.57**	<.0001	**0.50**	<.0001	
W7	**0.72**	<.0001	**0.73**	<.0001	0.67	.0189	
							Humerus
U1	**4.33**	<.0001	**4.44**	<.0001	**4.15**	<.0001	
U2	1.20	.0473	1.24	.0166	1.17	.4913	
U3	**4.42**	<.0001	**4.53**	<.0001	**4.23**	<.0001	
U4	1.24	.0198	**1.28**	.0059	1.20	.4082	
U5	0.85	.0744	0.88	.1470	0.77	.2521	
U6	0.91	.2899	0.94	.4870	0.83	.3973	
U7	0.90	.2143	0.92	.3769	0.81	.3600	
							Clinical vertebral
V1	**0.10**	<.0001	**0.09**	<.0001	**0.14**	<.0001	
V2	**0.17**	<.0001	**0.16**	<.0001	**0.25**	<.0001	
V3	**0.28**	<.0001	**0.28**	<.0001	**0.33**	<.0001	
V4	**1.70**	<0001	**1.70**	<.0001	**2.06**	<.0001	
V5	0.79	.0111	**0.80**	.0057	0.92	.6249	
V6	**0.75**	.0020	**0.77**	.0007	0.86	.3884	
V7	**0.18**	<.0001	**0.18**	<.0001	**0.21**	<.0001	
V8	**0.33**	<.0001	**0.33**	<.0001	**0.41**	<.0001	

Note: bold values are statistically significant at =.01; See Table 1 for a description of the case definitions.

Subsequent analyses of the hip fracture data when long-term care residents were excluded revealed similar RR estimates to those reported in Table 3. For example, when the data for males and females were combined, case definitions H5 (RR=1.44; *p*<.0001), H6 (RR=1.39; *p*<.0001) and H7 (RR=1.37; *p*=.0002) resulted in over-ascertainment of hip fracture rates.

For wrist fractures, most definitions resulted in under-ascertainment of fracture rates except for W1 and W3 in the combined and female populations and W4 and W6 in the male population. Among the seven definitions investigated for humerus fractures, U1 and U3 over-ascertained incidence rates in the combined, female, and male populations while the remaining case definitions did not result in significant differences from the CaMos data. Of the eight case definitions investigated for incident clinical vertebral fracture; six resulted in under-ascertainment in the overall and male populations and seven resulted in under-ascertainment in the female population. One case definition resulted in over-ascertainment and one resulted in no significant difference from the CaMos data in the combined population.

**Figure 1:**
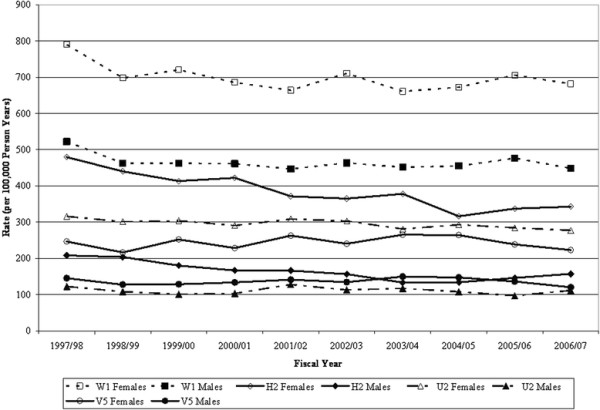
Trends in Crude Osteoporosis-Related Incident Fracture Rates for Selected Administrative Data Case Definitions, 1997/98 ‒ 2006/07.

Crude annual fracture rates, stratified by sex, for the case definitions selected as the references for the GEE models, H2, W1, U2, and V5 are reported in Figure 1. These case definitions resulted in the identification of 81187 incident fractures in the study period, of which 26.2% were hip, 39.4% were wrist, 18.2% were humerus, and 16.2% were vertebral fractures. The year x definition interaction was statistically significant for hip (*p*<.0001), but not for wrist (*p*=.4259), humerus (*p*=.3515), and vertebrae (*p*=.0865). For hip fracture, while H2 showed an average annual decrease of 4.1%, six case definitions resulted in smaller estimates of decline over time (*p*<.01): H4, H5, H6, H11, H12, and H13.

Tests of the year main effect revealed a decreasing trend for wrist (*p*<.0001), an increasing trend for vertebral (*p*=.0012), and no statistically significant change for humerus (*p*=.2226). For wrist fractures, all case definitions with the exception of W3 resulted in incidence estimates that were significantly lower than then reference definition (*p*<.0001). For humerus fractures, all case definitions resulted in incidence estimates that were significantly different from the reference definition (*p*<.0001) with the exception of U4 (*p*=.2315). The same finding was observed for vertebral fractures with the exception of V6 (*p*=.0555).

## Discussion

This study adopted a population-based methodology to assess the validity of multiple case definitions for ascertaining osteoporosis-related incident fractures in administrative data. Fracture rates estimated from hospital and physician billing claims data were compared to clinically-validated fracture estimates obtained from a representative sample of the Canadian population. In addition, estimates from multiple administrative data case definitions were compared over time.

For hip fracture, the magnitude of under- or over-ascertainment compared to CaMos data was modest regardless of the choice of case definitions. This suggests that administrative data are a valid data source for ascertaining cases of hip fracture, which is consistent with the findings of previous studies [3,15,40]. However, there were sex-specific differences in the magnitude of over- or under-ascertainment. Only three hip case definitions did not result in over-ascertainment in females. For males, these same case definitions resulted in under-ascertainment of fracture rates. We hypothesize that sex-specific differences in hip fracture inpatient treatment, which have been attributed to differences in comorbidity characteristics of males and females at the time of hip fracture [41], contributed to these findings. Alternatively, there may be sex-specific differences in the reporting of hip fracture in the CaMos data.

The magnitude of the RR estimates for wrist, humerus, and vertebrae case definitions were generally similar for males and females, suggesting there may be fewer differences in the treatment of these fractures in the health care system. Case definitions that required a diagnosis in two or more physician claims often resulted in under-ascertainment of wrist fractures. For chronic conditions such as diabetes and inflammatory bowel disease, the presence of a single diagnosis code may indicate an attempt by a physician to ‘rule-out’ the condition in a patient [23] and produce a case definition with an excess of false positives. However, this rule-out effect does not appear to be evident for fractures, indicating that individuals may not have multiple visits to physicians with a diagnosis code for one of these fractures. The amount of over-ascertainment of fracture rates when a fracture-free period for incident cases was not adopted varied with the fracture site; it was large for humerus and vertebrae fractures but small for wrist fractures. For incident clinical vertebral fractures, the study findings are consistent with previous research showing that fractures may be under-ascertained in administrative data [14], even if both hospital and physician data are used to construct the case definition. Health care professionals in both in-patient and out-patient settings cannot reliably diagnose these fractures.

The magnitude of change in incidence estimates over time varied across the case definitions for hip, but not for the remaining fracture sites. Further investigation revealed, however, that this difference was not due to the transition from ICD-9-CM to ICD-10-CA coding in hospital data. When we tested the sex-specific differences in the rate of change in hip fracture estimates before and after the introduction of ICD-10-CA, no statistically significant differences (*p*<.01) were observed.

This study has some limitations. Fracture case definitions were not validated using medical chart review, a method that has been recommended and used in previous studies [14]. Chart-based validation of fracture case definitions based on both hospital and physician claims databases would be difficult in Manitoba’s universal health care system because patients may receive care from more than one clinic, facility, or physician group, which could potentially result in multiple charts. Also, chart review may result in low power to validate case definitions in population sub-groups and for low-incidence events and the generalizability of the findings will be influenced by the method of sample selection. A second limitation is that we compared aggregate fracture estimates and tested whether there was a significant difference between the administrative data case definitions and the CaMos case definitions rather than whether they were equivalent. A test of statistical equivalence may have been preferred, but to our knowledge, no such test has been developed for count data [42]. The appropriateness of using CaMos data as a reference standard for evaluating the validity of fracture case definitions may be questioned, because the sample represents the Canadian population but not necessarily the Manitoba population. However, previous research has demonstrated that for hip fracture, Manitoba incidence estimates do not differ from national estimates [43]. Furthermore, self-report instruments have been shown to result in under-ascertainment of hip fractures when compared to administrative data [30,44]. Virnig et al. found that patient factors such as living with others, and mental status as identified from the mini-mental status exam score, and being unable to stand without using one’s arms were associated with a false negative hip fracture self-report [44]. However, it is important to note that CaMos uses both self- and interviewer-administered instruments to capture comprehensive information on fracture history.

## Conclusions

In summary, a number of recommendations for ascertaining osteoporosis-related fractures from administrative data arise from this study. For hip fractures, different case definitions for females and males might be considered, with a primary diagnosis of hip fracture and service codes recommended for ascertaining female cases. For wrist fractures, service codes should be used to construct the case definition. For both humerus and clinical vertebral fractures, the use of a fracture-free period is important to ascertain incident events. Overall, ascertainment of incident fracture cases does not require the use of more than one record with the relevant diagnosis code. The findings demonstrate that administrative data are generally useful for establishing a surveillance program about osteoporosis-related fracture.

## Competing interests

William Leslie: Speaker bureau and unrestricted research grants: Merck Frosst. Research honoraria and unrestricted educational grants: Sanofi-Aventis, Procter & Gamble. Unrestricted research grants: Novartis, Amgen. Unrestricted educational grants: Genzyme. Advisory boards: Genzyme, Novartis, and Amgen. Lisa Lix: Unrestricted research grant: Amgen Canada Ltd. Suzanne Morin: Consultant to: Procter & Gamble, Sanofi-Aventis, Servier, Amgen, Novartis. Speaker bureau: Procter & Gamble, Sanofi-Aventis. Unrestricted research grant: Amgen Colleen Metge: Unrestricted research grant: Amgen Canada Ltd Patricia Caetano: Unrestricted research grant: Amgen Canada Ltd All other authors have no competing interests.

## Authors' contributions

LML planned the study and analyses and drafted the manuscript. MA and BAO extracted the data and conducted the analyses. PC, SM, CM, and WDL participated in planning the study and analyses. DG, NK, and JP participated in planning the analyses of the CaMos data and assisted in drafting the manuscript. All authors read and approved the final manuscript.

## Appendix: Physician service codes used to identify osteoporosis-related fracture cases

## A. Services codes used to identify hip fractures

0865: fractures, lower extremity, femur, neck, closed reduction, cast or traction

0868: fractures, lower extremity, femur, neck, open reduction with internal fixation

0870: fractures, lower extremity, femur, neck, prosthetic replacement

0872: fractures, lower extremity, intertrochanteric, closed reduction

0874: fractures, lower extremity, intertrochanteric, open reduction

1149: joints, anthroplasty, hip, femoral head replacement type

1150: joints, arthroplasty, hip (cup or total hip replacement prosthesis)

1154: joints, arthroplasty, hip, femoral head, where previous prosthesis, etc. require removal

## B. Service codes used to identify wrist fractures

0807: fractures, upper extremity, distal end, closed reduction

0810: fractures, upper extremity, distal end, open reduction

0811: fractures, upper extremity, distal end, skeletal pinning

0821: fractures, upper extremity, radius and ulna, closed reduction

1851: plaster casts, forearm

1854: plaster casts, elbow to fingers

1856: plaster casts, hand to wrist

1860: plaster casts, shoulder to hand

## C. Service codes used to identify incident clinical vertebral fractures

7025: radiology, chest, posteroanterior and lateral

7035: radiology, spine and pelvis, spine, complete

7036: radiology, spine and pelvis, cervical spine, routine views

7037: radiology, spine and pelvis, spine, 2 full areas

7039: radiology, spine and pelvis, pelvis, anteroposterior view

7041: radiology, spine and pelvis, sacroiliac joints

7054: radiology, lumbo-sacral, routine views with special added views (obliques and/or flexion)

7061: radiology, spine and pelvis, single combining region (thoraco-lumbar)

7193: radiology, spine and pelvis, lumbo-sacral, routine views

7194: radiology, spine and pelvis, thoracic spine

7224: radiology, computerized axial tomography thorax exam

7225: radiology, computerized axial tomography abdomen and/or pelvis exam

7228: radiology, computerized axial tomography spine-thoracic exam

7229: radiology, computerized axial tomography spine-lumbar exam

7331: radiology, chest, ribs, both sides

7332: radiology, chest, thoracic inlet, two views

7339: radiology, spine and pelvis, pelvis with lateral hip joint

7341: radiology, skeletal survey (thorax, skull, thoracic and lumbar spine, pelvis, long bones)

7402: radiology, spine and pelvis, special views (minimum two views)

7519: MRI, limited spine, one segment multi-slice t2 (one or two echoes)

7520: MRI, limited spine one segment multi-slice i.r. or t1

7521: MRI, limited spine one segment repeat (another plane, different pulse sequence to a max of two repeats)

7522: MRI, intermediate, spine, 2 adjoining segments multi-slice t2 (one or two echoes)

7523: MRI, intermediate, spine, two adjoining segments multi-slice i.r. or t1

7524: MRI, intermediate spine, two adjoining segment repeat (add plane, different pulse sequence, maximum two repeats)

7525: MRI, complex spine, two or more non-adjoining segments, multi-slice t2 (1 or 2 echoes)

7526: MRI, complex spine, two or more non-adjoining segments, multi-slice i.r. or t

7527: MRI, complex spine, two or more non-adjoining segment repeat (add plane, different pulse sequence, maximum two repeats)

## Pre-publication history

The pre-publication history for this paper can be accessed here:

http://www.biomedcentral.com/1471-2458/12/301/prepub
